# Phenolic Acid–β-Cyclodextrin Complexation Study to Mask Bitterness in Wheat Bran: A Machine Learning-Based QSAR Study

**DOI:** 10.3390/foods13132147

**Published:** 2024-07-06

**Authors:** Kweeni Iduoku, Marvellous Ngongang, Jayani Kulathunga, Amirreza Daghighi, Gerardo Casanola-Martin, Senay Simsek, Bakhtiyor Rasulev

**Affiliations:** 1 Department of Coatings and Polymeric Materials, North Dakota State University, Fargo, ND 58102, USA; 2 Biomedical Engineering Program, North Dakota State University, Fargo, ND 58102, USA; 3 Cereal Science Graduate Program, Department of Plant Sciences, North Dakota State University, Fargo, ND 58102, USAssimsek@purdue.edu (S.S.); 4 Department of Multidisciplinary Studies, Faculty of Urban and Aquatic Bioresources, University of Sri Jayewardenepura, Gangodawila, Nugegoda 10250, Sri Lanka; 5 Whistler Center for Carbohydrate Research, Department of Food Science, Purdue University, West Lafayette, IN 47907, USA

**Keywords:** β-cyclodextrin, flavors, binding affinity, machine learning, QSAR

## Abstract

The need to solvate and encapsulate hydro-sensitive molecules drives noticeable trends in the applications of cyclodextrins in the pharmaceutical industry, in foods, polymers, materials, and in agricultural science. Among them, β-cyclodextrin is one of the most used for the entrapment of phenolic acid compounds to mask the bitterness of wheat bran. In this regard, there is still a need for good data and especially for a robust predictive model that assesses the bitterness masking capabilities of β-cyclodextrin for various phenolic compounds. This study uses a dataset of 20 phenolic acids docked into the β-cyclodextrin cavity to generate three different binding constants. The data from the docking study were combined with topological, topographical, and quantum-chemical features from the ligands in a machine learning-based structure–activity relationship study. Three different models for each binding constant were computed using a combination of the genetic algorithm (GA) and multiple linear regression (MLR) approaches. The developed ML/QSAR models showed a very good performance, with high predictive ability and correlation coefficients of 0.969 and 0.984 for the training and test sets, respectively. The models revealed several factors responsible for binding with cyclodextrin, showing positive contributions toward the binding affinity values, including such features as the presence of six-membered rings in the molecule, branching, electronegativity values, and polar surface area.

## 1. Introduction

In higher organisms, the taste buds detect flavors in the oral cavity wherein wheat and wheat bran products give off a sensation of bitterness [1]. The bitter flavor is a taste modality, and the perception of bitterness in wheat products could suggest the presence of phenolic compounds [2]. Experimental studies have concluded that phenolic compounds can bind to the oral cavity’s taste genes (i.e., TAS2R16). The consequence of this phenomenon is the resultant bitter sensation that individuals perceive [1]. The phenolic acids are produced by plants in response to oxidative stress and they consist of phenyl, hydroxyl, and carboxylic acid fragments [3]. They are classified into two subgroups: hydroxybenzoic and hydroxycinnamic acids [4], which give them a variety of characteristics mainly attributed to their chemical activity and applications [5,6]. In addition to their bio-based origin, most phenolic acids are safe for consumption in various food products. Most phenolic acids display hydrophobic characteristics, but some exhibit a spectrum of solubilities, depending on their chemical structure [7]. For example, gallic and p-coumaric acids are phenolic acids soluble in water [8,9]. They are also notorious for their capacity to form hydrogen bonds with other molecules that can influence their overall activity. However, this interaction can also affect the subsequent molecule’s characteristics. They can undergo reactions like oxidation, esterification, and conjugation, modifying their structure, chemical activity, biological properties, and stability [10]. The phenyl ring in the chemical composition of phenolic compounds plays a fundamental role in their antioxidative, anticancer, and antibacterial properties. They are considered very beneficial to human health [11]. The presence of phenolic acids in food can influence the perception of food; hence they are often extracted or broken down before consumptive processes [12]. In the case of the fermentation process, this involves the breakdown of molecules like phenols, polyphenols, and alkaloids, while solvent extraction involves using solvents like methanol, ethanol, and acetone to extract phenols from a sample of interest. Both methods mentioned are demanding and could raise health concerns due to the availability of solvent residuals or remnants. However, an alternative like cyclodextrin entrapment of phenolic compounds could reduce these drawbacks [13]. In previous studies, this process has shown several benefits, including improved kinetics, bioavailability, stability, and solubility of biomolecules. In summary, these oligosaccharides can form inclusion complexes with several compounds with distinctive hydrophobic characteristics [14].

Cyclodextrins are cyclic oligosaccharides that are non-toxic derivatives of starch compounds. Complexing molecules with cyclodextrin is a process that has proven effective in applications like propagating polymers, improving solubility, and providing bioavailable environments for insoluble compounds. Additionally, entrapping ligands within cyclodextrins is a common practice for influencing the perception of flavonoids [15,16,17]. The three most commonly used forms of cyclodextrin compounds are: α-, β-, and γ- cyclodextrins. They are differentiated based on their different sizes; the oligosaccharide with the smaller ring is α-, the β- is mid-sized, and the larger oligosaccharide is γ-cyclodextrin. They are derived from bio-based enzymatic activity and are safe for consumption, as FDA-funded studies have declared. Their sizes are relative to their application and ability to encapsulate the molecules within their cavities [18]. It is important to mention that, among all three cyclodextrins, the β-cyclodextrin variant has shown promising characteristics related to cavity dimensions, non-toxicity, and other aspects like the ability to manage specific reactions, the formation of complexes, and solubilizing and stabilizing molecules [19]. Additionally, β-cyclodextrin is statistically the most commonly occurring cyclodextrin, and it is very affordable and easy to use [20,21,22,23,24,25,26].

Computational approaches have come to be widely applied in the pharmaceutical industry, food industry, and in materials science. Thus, quantum-chemical and machine learning (ML)-based quantitative structure–activity relationship (ML/QSAR) methods are routinely applied to investigate the properties of various biologically active compounds and materials [27,28,29,30,31,32,33]. Moreover, nowadays the combination of two or more methods is becoming more popular and beneficial in the design of compounds/materials and explanation/prediction of the underlying interactions in chemical systems [34,35,36]. For example, in regard to cyclodextrins and their interactions with other compounds, a study conducted by Mirrahimi et al. uses combined molecular docking and quantitative structure–property relationship (QSPR) models with the aim of predicting the stability constant attained when a guest molecule is encapsulated within a β-cyclodextrin, as well as aiming to understand the underlying interactions between the guest ligands and the host β-cyclodextrin molecule [37]. In another work by Antonio et al. [38], the authors attempted to understand the interaction mechanisms of stabilizing olive biophenols (OBPs) in β-cyclodextrins for the masking of sensations of bitterness. This study employed molecular dynamics to observe the polar and hydrophobic interactions of different OBPs in β-cyclodextrin. Furthermore, Tuba et al. combined experimental and computational studies to unveil the main interactions related with the binding of phenolic acids—β-CD [39]. For this purpose, the authors used a computational process that involved molecular docking of phenolic acids into the molecular cavity of β-cyclodextrin, followed by the development of a QSPR model. Additionally, their study was based on the notion that complexing phenolic acids with β-cyclodextrin would enable the masking of bitterness by taste receptors. In this case, the authors only performed the encapsulation studies for three phenolic acid compounds [39]. Therefore, and following this idea, we carried out a broader study using 20 phenolic acid compounds of interest, which were docked into β-cyclodextrin to generate more reliable ligan–host complexes and obtain different binding constant values, in order to assess the stability of the complexes. Afterward, these response values were connected to ML/QSPR models using topological, topographical, semi-empirical, and quantum-chemical descriptors from the ligands to identify the factors that are related the most to the activities under consideration.

## 2. Materials and Methods

### 2.1. Dataset Preparation

The chemical structures of the 20 known single phenolic acids were built using ChemAxon [40] and Marvin Sketch version 22.13.0 [40]. In the first step, the β-cyclodextrin–ligand complexes were built by applying Avogadro [41] and HyperChem 8.0 software [42]. Then, the structures were optimized using the Bio-CHARMM force field which is equivalent to CHARMM27 for the case of biomolecules [43]. The final structures of the complexes were investigated by Auto Dock Vina 1.2.0 [44] software to perform molecular docking calculations.

The optimized ligand binding conformations were obtained, as well as all the necessary data for correlating phenolic acid activity (binding affinities) to their physiochemical properties (descriptors). Additionally, AutoDock Vina served as the source of the binding score values, and related scholarly articles served as sources for experimental activity data (binding affinities) [39,45,46,47,48,49,50,51]. Table 1 depicts the name of the ligands used in this study together with additional computational properties.

After importing upright and inverted structures into the Avogadro software 1.95 [41], the necessary corrections were implemented. Explicit hydrogens were added to investigated structures during the docking process on AutoDock Vina. Afterward, the structures were visualized by PyMol to highlight the interactions between the ligand and β-cyclodextrin and ensure a net charge of zero of the complex.

### 2.2. Binding Constant Calculations

The binding affinity was calculated by employing the PM6 semi-empirical QM energy model in MOPAC 2012 software [52]. MOPAC is a computational chemistry software to calculate the quantum-chemical and thermodynamic properties of molecules. It contains a set of semi-empirical methods, like AM1, PM3, PM6, and MNDO. The listed methods can be used to calculate the electronic properties, spectral properties, reaction mechanisms, transition states, reactivity, and solubility of molecules. As stated, the PM6 method was selected as the most functional for its reputation in terms of accuracy in calculating molecular and quantum-chemical properties. Additionally, the computational accuracy of PM6 is comparable to experimental conditions and findings, with some exceptions [53]. Log units of binding affinity were used as the chemical activity or response variable in the second model and log binding energy in the case of the third model. JMOL 14 software was used to visualize the output files from MOPAC calculations [54]. It pictures molecular orbitals and electron densities, and the 3D rendering of molecular structures.

### 2.3. Docking Procedures

Auto Dock Vina 1.2.0 software was selected for the molecular docking and scoring calculations. It performs grid-based docking, flexible ligand-based docking, and multiple docking/ligand screening procedures [44]. The output analysis of the docking study was carried out with PyMol [55] to visualize and investigate the intermolecular interactions of docked molecules. As discussed earlier, the most convenient docking method was employed: the rigid receptor and flexible ligand approach. Afterward, AutoDock Vina was used to create optimized versions of the ligand conformation within the cavity of β-cyclodextrin and output the different binding affinities of each conformation. From this point, the two best ligand conformations with the highest binding affinity scores were selected. Each chosen conformation has either an upright or inverted conformation to the β-cyclodextrin. To be considered upright, most of the body of the ligand must be facing the position of the β-cyclodextrin, which has two hydroxyl groups extending from the ends of each glucose subunit. To be considered inverted, most of the body of the ligand must face the β-cyclodextrin position with one hydroxyl group extending from the ends of each glucose subunit. The upright and inverted complexes were collected for each ligand to see which has the better binding affinity and more substantial molecular interactions.

### 2.4. Molecular Descriptors Generation

In the next step, Dragon 6.0 software was used to generate geometrical, topological, and constitutional descriptors for further ML/QSAR analysis [56]. Dragon 6 is software used to calculate different types of molecular descriptors, varying from zero to 3-dimensionality (0D to 3D). However, due to the limitations of the software, the inclusion complexes were not used for descriptor generation and only ligands from each inclusion complex were considered in the generation of the molecular descriptors. In a subsequent filtering, all the invariant and close-to-constant descriptors were discarded, keeping only 1200 descriptors of the investigated ligands (phenolic acids).

Herein, we collected quantum-chemical descriptors to combine them with other descriptors obtained from the abovementioned Dragon software for further GA-MLR analysis. The quantum-chemical PM6 method in MOPAC provides the desired level of accuracy in calculating the selected quantum-chemical descriptors for further use in developing QSAR models. The following quantum chemical descriptors were the main ones calculated: heat of formation (HF) and molecular orbital energies like HOMO/LUMO energies. The orbitals (HOMO and LUMO) were evaluated in JMOL and further documented.

### 2.5. Variable Selection and QSAR Modeling

After generating the descriptors, the set of 20 phenolic acid compounds was split in a ratio of 4:1 (80–20%) into training and test sets, respectively. This was carried out by sorting the binding constants for each study in ascending order by binding activity, and hence assigning the fifth compound to the test set. As a result, 16 compounds were included in the training set and 4 compounds in test set.

QSARINS 2.2.4 software was applied to perform the feature selection and model selection using the MLR and GA procedures with the aim of optimizing the selection of descriptors to develop predictive models. Additionally, predictive models contain the combination of descriptors that are best at predicting the studied property.

Feature selection in QSARINS aims to find the best combination of descriptors which leads to highly performing and accurate ML models. In this study the preliminary feature selection based on GA-MLR within the QSARINS 2.2.4 was implemented [57,58,59,60,61]. In this work, the selection process was started with 500 random models and executed 3000 iterations of evolution. Additionally, a mutation rate of 0.35 was assigned for the GA feature selection process.

The GA algorithm assigns each descriptor a genome translated or encoded to the solutions generated. It creates a generation of descriptors that includes all sets of possible combinations. Beginning from generation 0, it is contingent on randomness to develop a viable solution. A fitness function is then applied, which evaluates how good each solution is. Solutions with higher fitness scores are then used as parents to generate new solutions based on the mutation rate parameter. After applying a crossover function on these parent solutions, GA creates solutions for the next generation, and this cycle is repeated depending on the number of generations requested. A considerable degree of randomness drives the selection and crossover processes. Top solutions with top scores per generation are chosen and moved to the next combinatorial generation. Then, a mutation is applied to change the randomness of a combination based on a probability function. The process is repeated as a closed loop until the number of generations provided has been satisfied or until it attains a solution to the sorting problem (best combination of descriptors).

As discussed above, the ML/QSAR approach aims to generate a mathematical relationship between an activity (chemical, biological, or physical) and a set of descriptors (molecular properties) that are best associated with this activity. Once a model is obtained and validated, this relationship/model is used to predict the activity of other molecules within the applicability domain, which is based on the set of molecules used to generate the model. The quality of the models is assessed to obtain robust models and hence reliable predictions that are gathered from them, and to obtain different external and internal validations for the robustness and stability of the QSAR model. The cross-validation technique “leave-one-out” was used in the internal validation process of the QSAR models obtained from the feature selection of GA-MLR. This procedure consists of removing one molecule at a time from the training set and re-running the selected model against the individual molecules $(Q^{2}$*_LOO_)*. Later, the observed response values and the predicted response values calculated by the models are used to obtain the correlation coefficients *R*^2^ (Equation (1)) and the root mean square errors *RMSE* (Equation (2)) as statistical parameters to prove the performance of each model. This process is carried out for the training, cross-validation, and the external set (*R*^2^*_training_*, *Q_LOO_*, *R*^2^*_test_*).

(1) $$R^{2}=1-\frac{\sum_{i=1}^{n}{{(y}_{i}^{obs}-y_{i}^{pred})}^{2}}{\sum_{i=1}^{n}{{(y}_{i}^{obs}-{\tilde{y}}^{obs})}^{2}}$$

(2) $$RMSE=\sqrt{\frac{\sum_{i=1}^{n}(y_{i}^{obs}{-y_{i}^{pred})}^{2}}{n}}$$

where: *y_i_^obs^* is the experimental (observed) value of the property for the *i*_th_ compound; *y_i_^pred^* is the predicted value for *i*_th_ compound; *ỹ* is the mean experimental value of the property in the training and/or validation set, respectively; and *n* is the number of compounds in the training and/or validation set, respectively.

Furthermore, all descriptors were normalized by using MATLAB 2020 software [62], and the Python matplotlib library was used to generate detailed plots of our models for analysis [63].

### 2.6. Applicability Domain

Additionally, to ensure the reliability of our predictions, we employed a leverage approach to calculating the chemical applicability domain (AD) [64,65]. The Williams plot is the most common statistical tool in visual applicability domains. In this study, the Williams plot encompasses standard cross-validated residuals (RESs) of the data set, plotted against the leverage values (Hat Diagonal, h) of the data set. The residuals vs. the leverage plot efficiently capture response outliers (Std. residuals) and structurally influential compounds (Leverage) within the data set. Three sigma plots are graphical interpretations employed to ascertain outliers within the studied data set. Three sigma plots were used to detect molecules with response variables that are significantly different from the rest of the data set. The axis of the Std. residuals shows the standard deviation of the residuals multiplied by three (3σ). The h* index and the 3-sigma plot identify data points that carry too much weight by themselves and can skew the data distribution, i.e., the h* index is used to identify the data points that influence the QSAR model the most.

### 2.7. Descriptor Significance Plot

The significance plot is drawn directly from the QSAR model. As observed, the coefficient of each descriptor in the QSAR model represents the magnitude or influence of that descriptor on the model. Since these models are linear according to the MLR methodology, it is much easier to represent their magnitudes as bar plots. The coefficient sign could be positive or negative, and the magnitude determines the influence of a descriptor on a QSAR model.

## 3. Results and Discussion

In this work, we investigated the interactions of a set of 20 phenolic compounds with β-CD by estimating three different binding constants of the β-CD–ligand complexes. A molecular protein–ligand docking approach was used, followed by the ML/QSAR analysis. As a result, three ML/QSAR models were developed to describe and predict binding score affinity (Model 1), binding affinity (Model 2), and binding energy (Model 3), as shown in Table 2.

### 3.1. Binding Score Affinity (Model 1)

The best model for the prediction of the binding score affinity was obtained with three variables and is shown below in Equation (3):

***Log BSA*** = 0.078·***nR06*** + 0.353·***ATS4m*** − 0.294·***BEle3*** + 0.494 (3)

The statistical parameters of the model are depicted in Table 2 and the descriptors related to each model are shown in Table 3.

**Figure 1:**
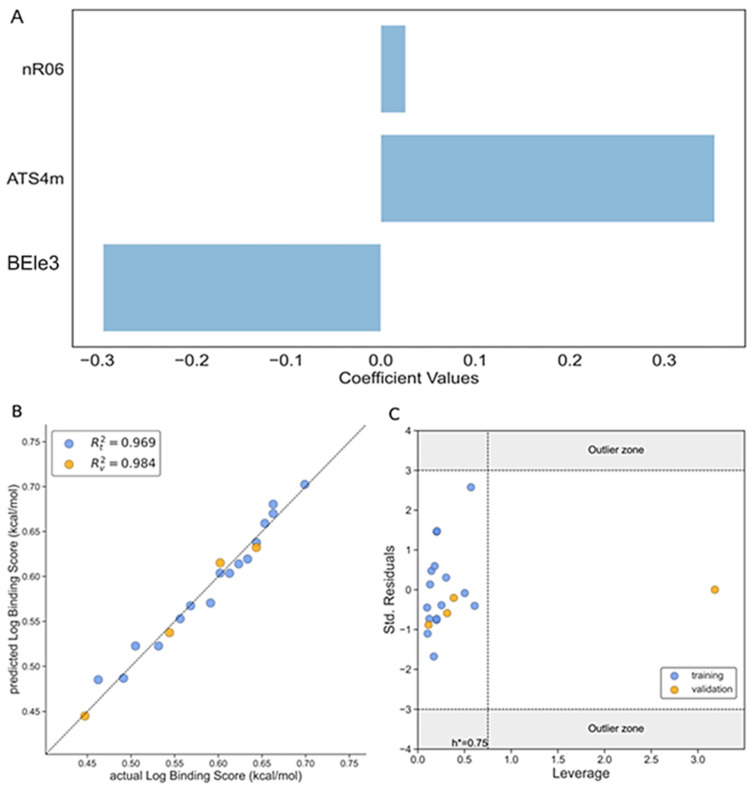
The performance of Model 1 according to Equation (3). (**A**) The magnitude of influence of different descriptors of the three-variable model on the binding score according to Equation (3); (**B**) the correlation graph between the observed and predicted values of *Log BSA*; (**C**) the Williams plot of standardized residual versus leverage of *Log BSA*. Training set (blue dots), test set (orange dots).

Figure 1A represents the influence of each descriptor on the log of binding affinity. From this graph it can be observed that the nR06 and ATS4m descriptors have a positive contribution to activity and the remaining descriptor, BEle3, has a negative impact to activity, with ATS4m showing the highest contribution of all the variables and nR06 the lowest one. It is important to highlight that in the Model 1 (Equation (3)) the correlation coefficient between the observed and predicted values R^2^ is 0.969 for the training set and 0.984 for the test set, as can be seen from Figure 1B.

The Williams plot in Figure 1C addresses the applicability domain regarding the three-sigma deviation and the leverage threshold h*. As can be seen from the figure, the majority of the molecules fall within the *(−3σ; +3σ*) domain and inside the h critical value, with only one compound falling outside h*. It implies that a leverage value exceeds the threshold value (h_i_ > h*). This result is mainly due to structural differences of this single molecule in our test set, which draws weight to itself and, at the same time, balances the model. It reflects the high leverage of this molecule but does not diminish the validity of this model.

**Figure 2:**
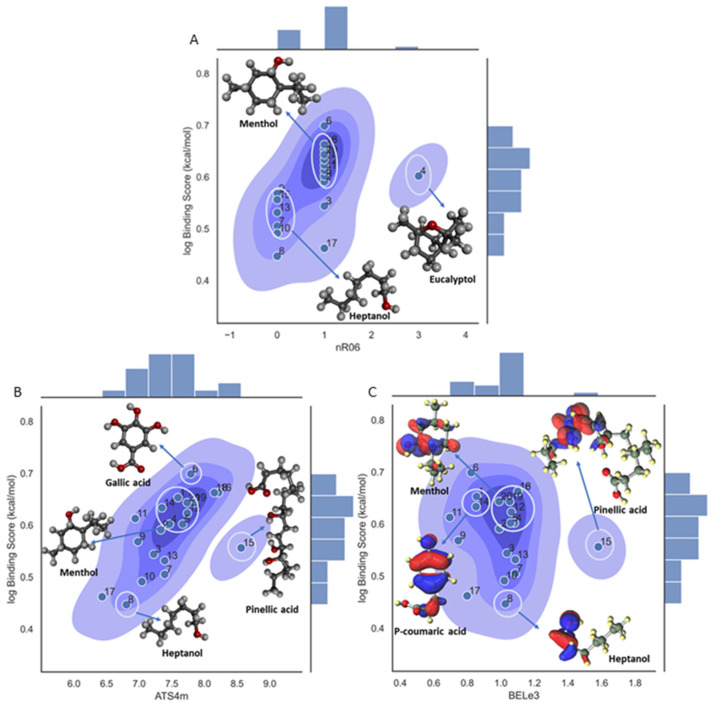
Density plot for the three-variable model regarding log binding affinity score, showing the molecular descriptor influence on the target property. (**A**) Density plot for the **nR06** descriptor; (**B**) density plot for the **ATS4m** descriptor; (**C**) density plot for the **BELe3** descriptor.

After confirming the validity and reliability of Model 1, in the next step a mechanistic interpretation of the model was also assessed by conducting a detailed analysis of the three descriptors involved in the model (see Figure 2A–C). For example, the nR06 descriptor has a small positive influence on the binding affinity in the model, the descriptor quantifies the number of six-membered rings in a molecule which belong to the class of fragment descriptors. In general, this molecular descriptor has a positive influence because of the π interactions that could stabilize the ligand within the β-CD pocket [66]. Moreover, as can be observed from Figure 2A, of all the structures with zero value for this descriptor, no-ring structures have most of the lower values for the binding affinity score, below 0.5 approximately, and, conversely, for ligands with one ring that have a log binding score affinity above 0.5. Also, it should be noted that eucalyptol (ligand 4), with three rings, has the highest value in all of the data but with a value around 0.6, and same is true for ligands 3 and 17, with log binding affinity scores not higher than expected. In these three cases this may have occurred because the model is multi-featured and this means the interactions with the other variables are significant as well.

The ATS4m descriptor is a 2D autocorrelation class descriptor, the Broto–Moreau autocorrelation of lag 4 (log function) is weighted by mass and is calculated from the molecular graph by summing the products of the atom weights of the terminal atoms of all of the paths of the considered path length (the lag). Therefore, there are two main aspects involved in the definition of this descriptor; the first is related to the frequency of the path. For the menthol and heptanol ligands there are 17 and 5 counted paths, respectively, which is connected to some extent to their branching. From this analysis, it can be noticed that an increase in branching positively impacts the log binding affinity score, as can be seen from Figure 2B, where a linear positive correlation is observed for most of the cases. This finding supports the idea that with higher branching more hydrogen interactions can occur between the ligands and the β-CD.

The third descriptor, BELe3, is a Burden eigenvalue descriptor and, as can be seen from Figure 2C, there is no linear tendency within the values in the range 0.8 to 1.2, and only ligand 15 is deviates from this with an extreme value of about 1.6 and with a binding affinity in the intermediate range.

Although the functionality of the BEle3 descriptor is ambiguous, there are some distinctive observations within the clusters. Molecules with a phenyl group or six-membered rings cluster together and tend to be higher on the binding affinity scale. Another observation is that the hydroxyl branches of the phenyl are favored over the methoxy branches. Additionally, shorter stem (hydroxybenzoic) molecules are favored over the longer stem (hydroxycinnamic) molecules. Gallic acid is of the hydroxybenzoic class and has three hydroxyl group branches, a short stem leading to carboxylic acid, and no methoxy groups. It has the highest binding affinity so far. In Figure S2A (the electrostatic potential graph), gallic acid shows more saturation of the electronegative charge than Vanillic acid (Figure S2B). This observation could be associated with the presence of the number of hydroxyl groups and is related to their electronic orbitals. As seen in Figure S2A,B, both molecules are readily reactive in a complex with BCD and retain their HOMO and LUMO orbitals (Figure S1A,B). Figure S1C shows maltol with a six-membered ring and the figure shows that it retains the HOMO and LUMO orbitals in the complex, which means it is readily reactive. Conversely, Figure S2D shows that menthol has more saturated positive and neutral charges and it does not retain its HOMO and LUMO orbitals (Figure S1D).

### 3.2. Binding Affinity (Model 2)

**Figure 3:**
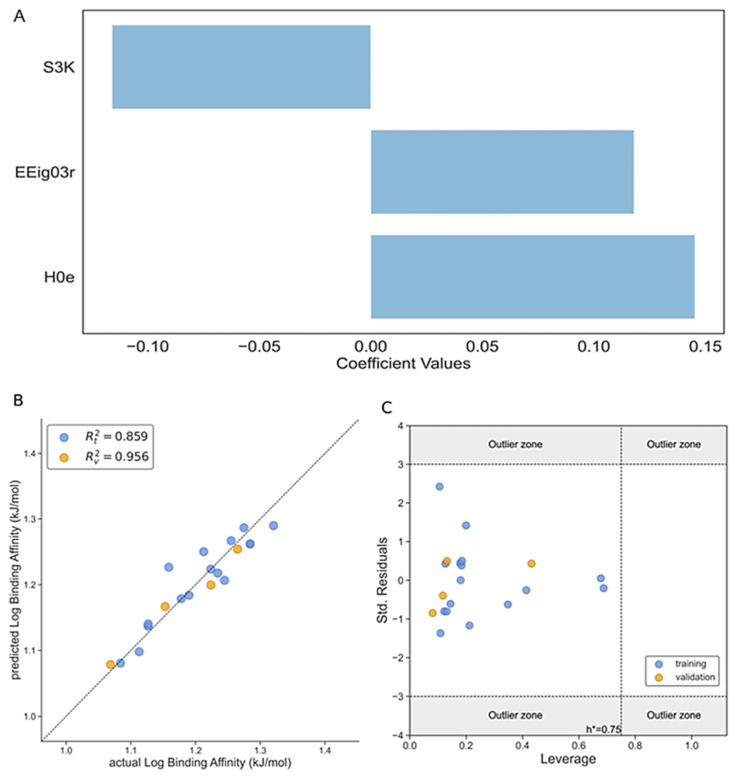
The performance of Model 2 according to Equation (4). (**A**) The magnitude of the influence of different descriptors of the three-variable model on the *Log BA* according to Equation (4); (**B**) the correlation plot between the observed and predicted values of *Log BA*; (**C**) the Williams plot of the standardized residual versus the leverage of *Log BA*. Training set (blue dots), test set (orange dots).

The second ML/QSAR model is related to the binding affinity (log BA, Table 2 and Table 3); a model with three variables was selected as the best one, with the EEig03r and H0e descriptors contributing positively to the binding constant values and S3K with a negative impact on the activity (see Equation (4)). It is important to remark that the H0e molecular descriptor has the highest impact on the response variable of our model, as is depicted in Figure 3A. The statistical parameters of Model 2 are shown in Table 2, which includes, among others, RMSE, MAE, and CCC, and all of them showed very good performance values—such as R^2^ = 0.859 for the training set and R^2^ = 0.956 for the test set. In Figure 3B, the actual vs. predicted values of the log binding affinity are plotted and good correlation is observed in both the training and test sets.

***Log BA*** = −0.116·***S3K*** + 0.127·***EEig03r*** + 0.145·***H0e*** + 1.064 (4)

Also, an applicability domain analysis of Model 2 was performed by the Williams plot, as shown in Figure 3C. As can be observed from this plot, all the ligands for both the training and test sets fall within the applicability domain range, which means they are inside the leverage critical threshold (h*) with values lower that h* = 0.75, and inside the (*−3σ; +3σ*) standard deviation.

**Figure 4:**
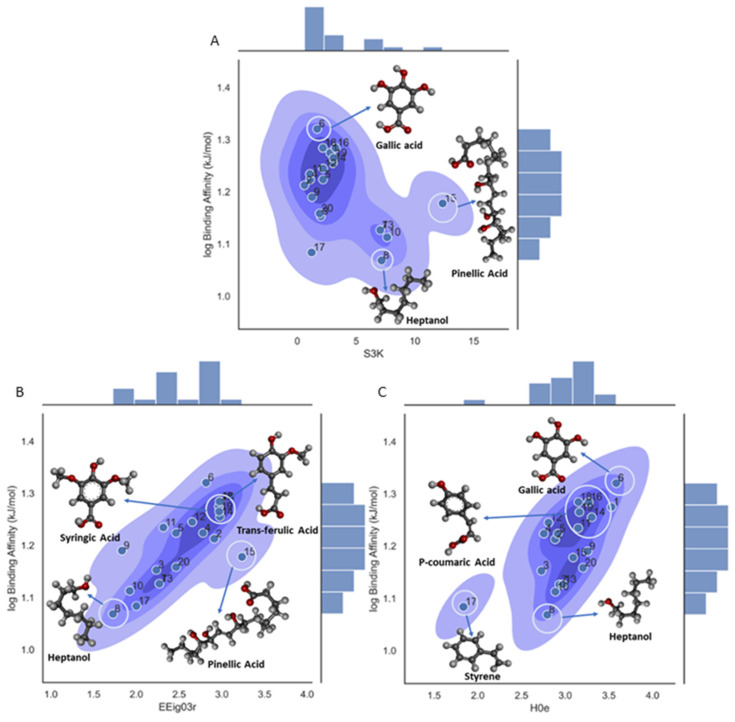
Density plot for the three-variable model on log binding affinity, showing the molecular descriptor’s influence on the target property. (**A**) Density plot for the **S3K** descriptor; (**B**) density plot for the **EEig03r** descriptor; (**C**) density plot for the **H0e** descriptor.

A deeper analysis on the influence of each descriptor and a mechanistic explanation were conducted for the three descriptors. The first descriptor, S3K, is the three-path Kier alpha-modified shape index. This molecular descriptor is a topological index that is related to branch centrality and encodes the number of paths with length k = 3 in an H-depleted molecular graph. As can be seen from Figure 4A, the ligands with aromatic rings have the lower descriptor values and the aliphatic chains have the higher ones. This is interesting because, although gallic acid (ligand 6) has a higher number of three-length paths, it has one of the lower values in the descriptor. This occurs because this descriptor (S3K) also considers the different shape contributions of heteroatoms and hybridization states, with the latter having lower values for aromatic rings and higher values for aliphatic chains. Other factors, such as the point of origination of a branch or the groups of a branch, could also influence the binding affinity. For example, Reinskje et al. showed that the binding affinity of p-alkylbenzamidinium chloride to serin proteinase trypsin27 could decrease with an increase in branching from the C1 carbon of the phenyl ring [67].

The second descriptor in the model, EEig03r, quantifies the topological complexity of a molecule based on the eigenvalues of the edge adjacency matrix of the molecule. This descriptor is mainly related to molecular branching. As can be seen in Figure 4B, the ligands that possess high values in EEig03r have a high degree of branching and high binding affinity values. In contrast, molecules with low values in EEig03r are more likely linear and have a less branched structure and lower binding affinity values, showing a clear positive trend related to activity. This could be related to the concept that branching could enhance interactions with β-CD. For example, is important to remark that there are similarities in the molecular structures of syringic (ligand 18) and trans-ferulic acids (ligand 19). Syringic acid has a binding affinity of −77 kJ/mol, and its structure shows four substituents in the phenyl ring. In the case of trans-ferulic acid, the phenyl ring has three substituting groups attached, and this could be a factor that is related to its high binding affinity value and hence its strong interaction with β-CD.

The third descriptor in the model, H0e, is defined as an H autocorrelation of lag 0/weighted by Sanderson electronegativity and classified inside the GETAWAY descriptor class. The term H is related to the leverage matrix, since this descriptor is calculated from the leverage matrix obtained by the centered atomic coordinates. Of the other terms, lag 0 refers to the topological distance with 0, i.e., only considering the atom itself and the last term ‘e’ is the weighting scheme, the electronegativity in the Sanderson scale for this descriptor. As can be observed from Figure 4C, there is a clear and positive correlation between the molecular descriptor and the binding affinity, with gallic acid (ligand 6) having the higher value in this descriptor as it has a high number of electronegative atoms (oxygen atoms) in the structure; five in total. Therefore, an increase in the total electronegativity of the molecule leads to an increase in the binding affinity value. The opposite case is shown for 1-heptanol (ligand 8) with only one oxygen in the structure, where a lower value in the descriptor leads to a lower value of the binding affinity. The increase in the total electronegativity of the molecules is also related to polarity and, therefore, the molecule will be more likely to participate in hydrogen bonding and other intermolecular interactions. The H0e descriptor could be related to the influence of the distribution of electronegative groups across the molecular topology and, consequently, those ligands with phenolic, carbonyl, hydroxyl, and carboxylic groups in their molecular structures should display a higher capacity to form hydrogen bonds and stronger interactions within the β-CD cavity.

### 3.3. Binding Energy

**Figure 5:**
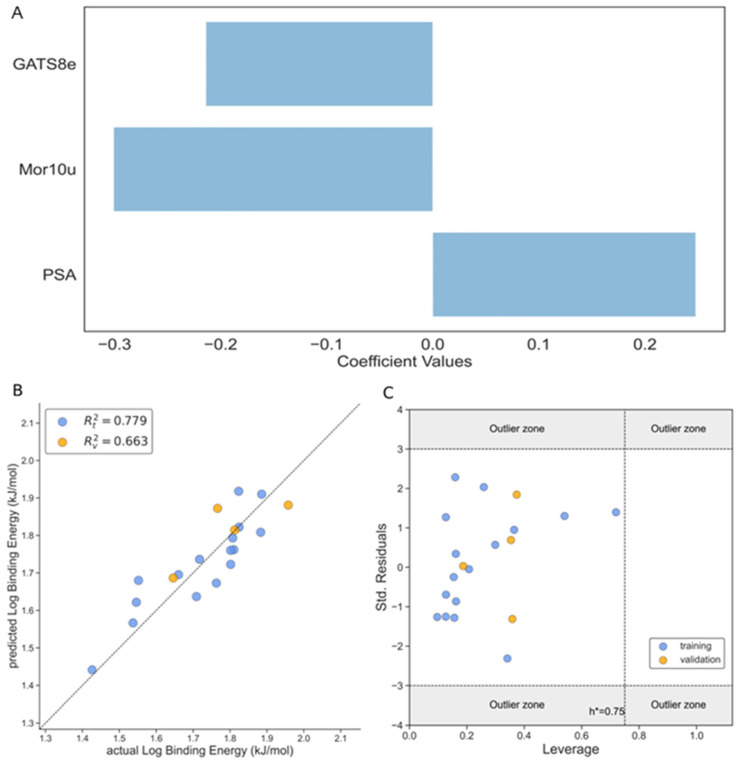
The performance of Model 3 according to Equation (5). (**A**) The magnitude of influence of the different descriptors of the three-variable model on the *Log BE* according to Equation (5); (**B**) the correlation plot between the observed and predicted values of *Log BE*; (**C**) the Williams plot of the standardized residual versus leverage of *Log BE*. Training set (blue dots), test set (orange dots).

The third model (Model 3) is developed for binding energy (BE) as a response variable. The model has three molecular descriptors that include TPSA, with a positive impact on activity, and GATS8e and Mor10u with a negative contribution to the binding energy, as can be observed from Equation (5) and Figure 5A. The quality of the model was validated with the test set and using the statistical parameters RMSE, MAE, R^2^, and others commonly used to prove the performance of such models. The R^2^ values for the training and test sets were 0.779 and 0.663, respectively, with adequate values for the other parameters for both the training and test sets (Table 3). Additionally, the actual vs. predicted values of the log binding energy are shown in Figure 5B for both the training and test sets.

***Log BE*** = −0.214·***GATS8e*** − 0.301·***Mor10u*** + 0.248·***TPSA*** + 1.798 (5)

The Williams plot shown in Figure 5C validates the model with the implementation of three sigma residuals and the leverage threshold h*. This figure shows that both the training and test sets fall within the three-sigma standard deviations and all the ligands fall inside h*, which implies that no leverage value is more significant than the leverage threshold value (h_i_ < h*).

**Figure 6:**
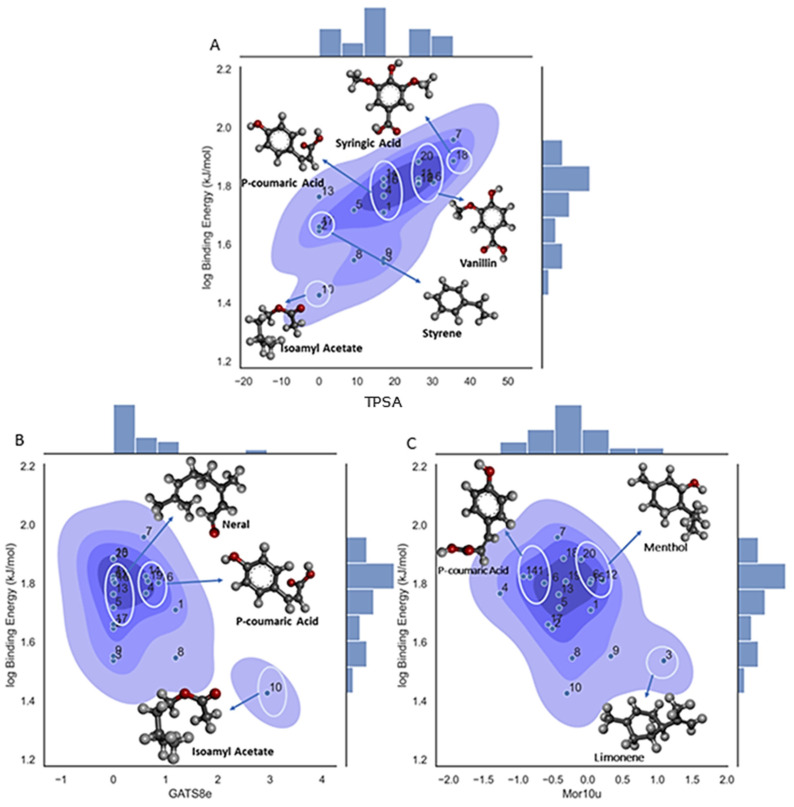
Density plot for the three-variable model of log binding affinity, showing the influence of the molecular descriptor on the target property. (**A**) Density plot for the **TPSA** descriptor; (**B**) density plot for the **GATS8e** descriptor; (**C**) density plot for the **Mor 10u** descriptor.

A set of important descriptors are included in Model 3. Thus, the PSA or TPSA descriptor is related to the topological polar surface area. This molecular descriptor is calculated based on the summation of tabulated surface contributions of polar fragments that includes the atoms involved and the bonding pattern (single, double, or triple bonds). These polar fragments are those containing oxygen and nitrogen and some that are ‘less polar’ with phosphorus and sulfur [68]. This explains the surface area of a molecule that is accessible to polar solvents. As can be seen from Figure 6A, there is a positive trend between the binding affinity and the molecular descriptor, with syringic acid (ligand 18) having the highest molecular descriptor value, corresponding to the high number of oxygen atoms in its structure; five in total for this compound, resulting in a high contribution to the total polar surface area calculations as described above and a high binding energy value. In the other case we have styrene (ligand 17) with no oxygen atoms in the structure and hence a low TPSA value. This tendency may occur because an increase in the number of oxygen atoms (TPSA) increases the occurrence of hydrogen bonds and hence stabilizes the ligand–β-CD interactions. In Figure 6B,C, the density plots for the remaining two descriptors in the model can be observed and, as can be seen, there is not a linear observable tendency among all of the values in the range, with only ligand 10 for both descriptors and ligand 3 for the Mor10u descriptor showing extreme values.

Although the functionality of the GATS8e descriptor is uncertain in Figure 6B, there are some observations that can be made on the pattern of clustering. One cluster contains molecules that were mostly hydroxycinnamic acids like sinapic, p-coumaric, and trans ferulic acid. Their common structural feature is that they possess three carbon stems on the C6-C3 side chain. The other cluster in the density graph contains maltol, gallic acid, menthol, and neral. The most common structural feature in the second cluster is the absence of a long side chain. Another cluster contains vanillic and syringic acid, which possess similar structural properties to the previous cluster. They are mostly hydroxybenzoic acids. It seems GATS8e segregated the structural difference between hydroxybenzoic acids and hydroxycinnamic acids. It can be noted that the cluster containing mostly hydroxycinnamic acids is higher than clusters containing hydroxybenzoic acids. From the significance graph, it is evident that the GATS8e descriptor has an inverse relationship with the binding affinity. The presence of the C6-C3 side group could impact the binding affinity adversely. It can affect the charge density, reducing hydroxycinnamic acids’ interactive strength during BCD entrapment.

In Figure 6C, the Mor10u descriptor has a distinctive clustering pattern. Molecules within the phenyl group are distributed away from zero. In the other cases, molecules with non-aromatic cyclic groups scored higher and are the furthest away from zero. Conversely, linear molecules have scores closer to zero. It seems that, in scoring molecules, the Mor10u descriptor separates the molecules based on intermolecular distances and 3D conformational analysis. Molecules like p-coumaric acid (14) and eucalyptol (4) have aromatic groups. Molecules like D-limonene (3), menthol (12), and hydroxymethylfurfural (9) have cyclic groups. Molecules like pinellic acid, isoamyl-acetate (10), and neral (13) are relatively linear. Additionally, in Mor10u, the molecules closer to zero have lower binding affinity values. The higher affinity molecules have phenyl groups or aromatic rings as common attributes. They are also clustered toward the upper half of the density graph. Most linear-structured molecules have a lower binding affinity and have descriptor scores close to zero. However, pinellic acid is linear but a member of the higher binding affinity cluster. This observation indicates that the size/molecular volume could also be a determining factor.

In this study, the predictive R^2^_train_ values for each ML/QSAR model (see Table) have the following order—Model 1 > Model 2 > Model 3, and show the proportion of variance within the binding affinity that is captured by the models. In this sense, the Model 1 suggests that it explains a larger portion of the variance within the response variable compared to Model 2 and Model 3. For the test set, the same tendency is observed for R^2^ and the other statistical parameters (RMSE, MAE, and CCC) that were used to prove the performance of our models.

## 4. Conclusions

In this study, we explored various structural and molecular mechanisms involved in interaction of phenolic acids with β-cyclodextrin, which can be correlating with masking bitterness in wheat bran-associated ligands. The applied computational approaches involve the combination of molecular docking and machine learning to unveil the mechanisms. Three ML/QSAR models were obtained with very good performances in both the training and test sets, where the model of the binding score affinity (BSA) showed the best performance with coefficients R^2^_train_ = 0.969 and R^2^_test_ = 0.984. The model has a descriptor which is the number of six-membered rings (nR06) that shows a positive influence on the binding constant and promotes stabilization through polar–π interactions. Another important descriptor, the 2D descriptor ATS4m, is related mainly to path frequency and shows a positive tendency towards the binding constant, and this descriptor is connected to molecule branching and hence increases the interactions between the ligand and the β-CD in the complex by different modes. Additionally, other descriptors, weighted by electronegativity values and topological polar surface area (TPSA), were able to show a positive influence on the binding constants in Models 2 and 3. This aligns with the outcomes that hydrophobic and/or lipophilic interactions drive most β-CD−Ligand interactions, highlighting the importance of solvation in a polar environment, like water, and also the relationship with hydrogen bonding interactions, as well as the instantaneous occurrences of dipole–dipoles. Other features that promote or hinder binding affinity are the atomic mass of the ligands and, understandably, the size of the ligands. Most of the ligands in this study fit well within the size range and become entrapped in the β-CD molecular pocket. These findings could be very helpful in designing new complex systems with better binding constant values and, hence, with improved bitterness masking properties.

## Figures and Tables

**Table 1:** Phenolic acids’ quantum-chemical properties and binding scores.

ID	β-CD-Ligand Complex	HOMO ^a^ (eV)	LUMO ^b^ (eV)	Gap (eV)	Binding Score (kJ/mol)	Binding Affinity (kJ/mol)
1	Caffeic acid-in	−9.1890	−1.0514	−8.1376	−4.5	−51.1831
2	Camphor-in	−10.1147	0.0704	−10.1851	−3.9	−34.4419
3	D-Limonene-in	−9.3119	0.0092	−9.3211	−3.5	−44.2580
4	Eucalyptol-in	−9.9785	0.1605	−10.139	−4.0	−52.1555
5	Eugenol-up	−8.9658	−0.1518	−8.8140	−4.0	−35.1691
6	Gallic acid-up	−9.5087	−1.2760	−8.2327	−5.0	−35.6459
7	Geranial-up	−9.5651	−0.2514	−9.3137	−3.2	−58.3673
8	Heptanol-up	−10.2024	−0.1426	−10.0598	−2.8	−26.6948
9	Hydroxy Methyl Furfural-up	−9.2240	−0.4026	−8.8214	−3.7	−64.9081
10	Isoamyl acetate-up	−10.1978	−0.3113	−9.8865	−3.1	−66.5904
11	Maltol-up	−9.8792	−1.3000	−8.5792	−4.1	−64.6218
12	Menthol-in	−10.1733	−0.0167	−10.1566	−4.2	−57.9313
13	Neral-up	−9.7932	−0.4443	−9.3489	−3.4	−66.7781
14	P-Coumaric acid-in	−9.3920	−0.3048	−9.0872	−4.3	−63.3347
15	Pinellic acid-in	−9.8072	0.0819	−9.8891	−3.6	−63.3198
16	Sinapic acid-up	−9.1111	−1.1979	−7.9132	−4.6	−90.6849
17	Styrene-up	−9.4684	−0.4950	−8.9734	−2.9	−45.7564
18	Syringic acid-up	−9.2231	−0.9025	−8.3206	−4.6	−76.8800
19	Trans Ferulic acid-up	−9.0647	−1.4466	−7.6181	−4.4	−64.1563
20	Vanillic acid-in	−9.1477	−0.9277	−8.2200	−4.4	−76.3647

^a^ Highest occupied molecular orbital. ^b^ Lowest unoccupied molecular orbital.

**Table 2:** Statistical parameter values for binding constant MLR models.

Parameters	Log BSA	Log BA	Log BE
Model # Number of variables	1 (Equation (3)) 3	2 (Equation (4)) 3	3 (Equation (5)) 3
*R^2^* (training set)	0.969	0.859	0.779
*RMSE* (training set)	0.0116	0.0256	0.0631
*MAE* (training set)	0.0095	0.0192	0.0527
*CCC* (training set)	0.985	0.924	0.876
*F*	126.902	24.349	14.117
*R*^2^ (cross-validation)	0.925	0.805	0.634
*RMSE* (cross-validation)	0.0182	0.0302	0.0812
*MAE* (cross-validation)	0.0135	0.0236	0.0698
*CCC* (cross-validation)	0.961	0.897	0.790
*R*^2^ (external test)	0.984	0.956	0.663
*RMSE* (external test)	0.0093	0.0156	0.0685
*MAE* (external test)	0.0082	0.0146	0.0563

**Table 3:** List of descriptors included in the QSAR models.

Descriptor	Description	Class
**Model 1—Binding Score Affinity**		
**nR06**	Number of 6-membered rings	Ring descriptors
**ATS4m**	Broto–Moreau autocorrelation of lag 4 (log function) weighted by mass	2D Autocorrelations
**BEle3**	Lowest eigenvalue No. 3 of Burden matrix/weighted by atomic Sanderson electronegativities	BCUT descriptors
**Model 2—Binding Affinity**		
**S3K**	3-path Kier alpha-modified shape index	Topological Indices
**EEig03r**	Eigenvalues	Edge adjacency indices
**H0e**	H autocorrelation of lag 0/weighted by Sanderson electronegativity	GETAWAY descriptors
**Model 3—Binding Energy**		
**GATS8e**	Geary autocorrelation of lag 8 weighted by mass	2D Autocorrelations
**Mor10u**	Signal 10/unweighted	3D-MoRSE descriptors
**TPSA**	Topological polar surface area using N,O polar contributions	Molecular properties

## Data Availability

The original contributions presented in the study are included in the article/supplementary material, further inquiries can be directed to the corresponding author.

## Supplementary Materials

The following supporting information can be downloaded at: https://www.mdpi.com/article/10.3390/foods13132147/s1, Figure S1, Title: Electronic orbitals of BCD-complexed molecules, showing the location of their HOMO-LUMO orbitals; Figure S2, Title: Electrostatic potential graph showing the saturation patterns of polar and non-polar groups in molecules.
