# Supplementary material for: Phenolic Acid–β-Cyclodextrin Complexation Study to Mask Bitterness in Wheat Bran: A Machine Learning-Based QSAR Study
Source: Foods. 2024 Jul 6;13(13):2147. doi: 10.3390/foods13132147 (PMC11241027; doi:10.3390/foods13132147)
Supplement: Supplementary file 1 [file foods-13-02147-s001.zip › foods-3041235-supplementary.pdf]

SUPPLEMENTARY DATA

Phenolic Acid – β-cyclodextrin Complexation Study to Mask Bitterness in Wheat Bran: A Machine-Learning-based QSAR Study

Kweeni Iduoku[1,2], Marvellous Ngongang[1], Jayani Kulathunga[3,4], Amir Daghighi[1,2], Gerardo Casanola-Martin[1], Senay Simsek[3,5], Bakhtiyor Rasulev[1,2*]

1 Department of Coatings and Polymeric Materials, North Dakota State University, Fargo, ND 58102, USA; kweeni.iduoku@ndsu.edu; gerardo.casanolamart@ndsu.edu; bakhtiyor.rasulev@ndsu.edu
2 Biomedical Engineering Program, North Dakota State University, Fargo, ND 58102, USA; kweeni.iduoku@ndsu.edu; amirreza.daghighi@ndsu.edu
3 Cereal Science Graduate Program, Department of Plant Sciences, North Dakota State University, Fargo, ND 58102, USA; jayani.maddakandaged@ndsu.edu
4 Department of Multidisciplinary Studies, Faculty of Urban and Aquatic Bioresources, University of Sri Jayewardenepura, Gangodawila, Nugegoda, Sri Lanka; jayani.maddakandaged@ndsu.edu
5 Whistler Center for Carbohydrate Research, Department of Food Science, Purdue University, West Lafayette, IN 47907, USA; ssimsek@purdue.edu

* Correspondence: bakhtiyor.rasulev@ndsu.edu

Table of Contents

Fig S1. Electronic orbitals of BCD-complexed molecules, showing the location of their HOMO-LUMO orbitals.

Fig S2. Electrostatic potential graph showing the saturation patterns of polar and non-polar groups in molecules

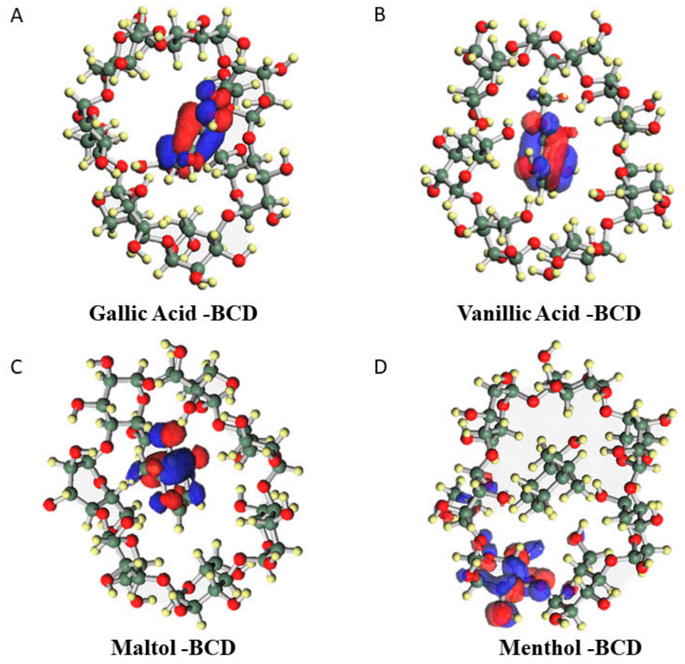

Figure S1: Electronic orbitals of BCD-complexed molecules, showing the location of their HOMO-LUMO orbitals.

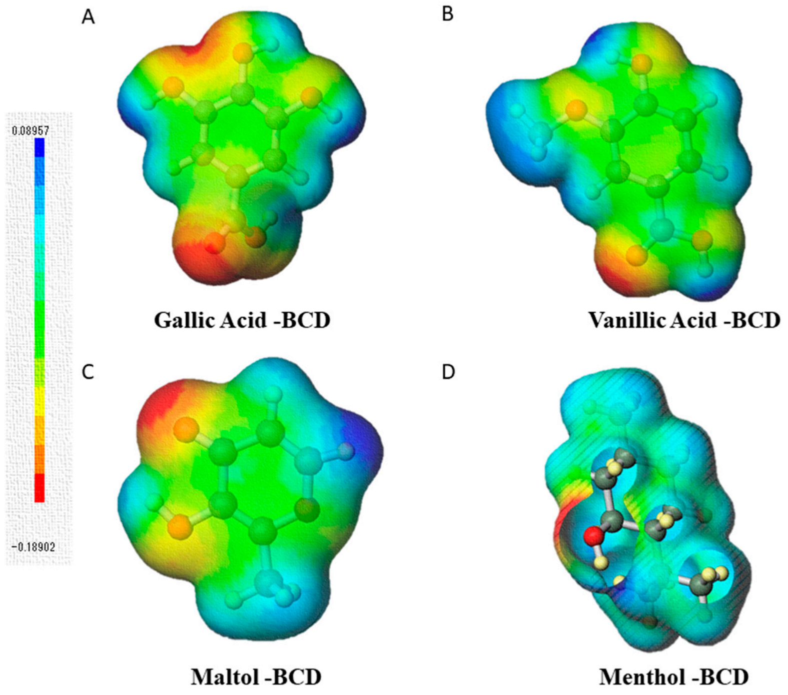

Figure S2: Electrostatic potential graph showing the saturation patterns of polar and non-polar groups in molecules
